# Alveolar Lymphangioma in Neonate: A Case Report With Immune Profile

**DOI:** 10.1111/ipd.13309

**Published:** 2025-03-14

**Authors:** Muhammad Aiman bin Mohd Nizar, Benedict Seo, Haizal M. Hussaini, Brendan Young, Alison M. Rich

**Affiliations:** ^1^ Department of Oral Diagnostic and Surgical Sciences, Faculty of Dentistry University of Otago Dunedin New Zealand; ^2^ Department of Craniofacial Diagnostic and Biosciences, Faculty of Dentistry Universiti Kebangsaan Malaysia Kuala Lumpur Malaysia; ^3^ Dental and Oral Health Services Wellington Hospital Wellington New Zealand

**Keywords:** alveolar lymphangioma, D2‐40 (podoplanin), immunohistochemistry, lymphangioma, neonates

## Abstract

**Background:**

Alveolar lymphangioma is a rare condition that commonly develops on the alveolar ridge of Black male neonates. It typically presents as a bluish, dome‐shaped swelling smaller than 1 cm on the posterior alveolar ridge of the mandible or/and maxilla, that allows it to be diagnosed clinically. Most previously reported cases of alveolar lymphangioma report spontaneous resolution, and biopsy is seldom performed unless the lesion becomes symptomatic or interferes with feeding.

**Case Report:**

We report a case of oral alveolar lymphangioma on the anterior maxillary alveolar ridge of an 11‐day‐old infant of non‐black descent. The lesion presented as a pedunculated, reddish, lobular mass in the anterior maxilla. Given its atypical presentation and the potential for feeding difficulties, an excisional biopsy was performed. The diagnosis was confirmed histologically through biopsy and supported by immunohistochemical staining.

**Conclusion:**

This case expands the understanding of alveolar lymphangioma by reporting it in a non‐Black neonate with an unusual location and appearance, emphasising the need for excisional biopsy to rule out other potential oral lesions, especially neoplasms.

**Summary**

- Why this paper is important to pediatric dentists
  ○ Alveolar lymphangioma should be considered as one of the differential diagnoses of lesions affecting the alveolar ridge of neonates.
  ○ The unusual clinical appearance of an alveolar ridge lesion in neonates, with or without potential feeding difficulties, warrants clinical intervention and the establishment of a definitive histological diagnosis.
  ○ This case marks the first reported instance of alveolar lymphangioma presenting as a pedunculated lobular lesion in the anterior maxilla region. Additionally, it is the first case of alveolar ridge lymphangioma that has been histologically confirmed through immunohistochemistry staining.

## 1 Introduction

Lymphangioma is a vascular malformation attributed to congenital lymphatic dysplasia rather than a true benign neoplasm. According to the current knowledge, the pathophysiology of lymphangioma involves a PIK3CA gene mutation along with overexpression of vascular endothelial growth factor (VEGF)‐C and VEGF receptor type 3 (VEGFR3) which localises within the lymphatic endothelial cells [1]. Lymphangioma affects about 1 in 5000 newborns, with most cases identified by the age of 2. The head and neck are the most commonly affected regions, although intraoral lymphangioma is relatively rare, accounting for 1.9%–11.6% of all benign soft tissue tumours diagnosed in the oral cavity [2]. Lymphangioma is classified into two types based on the size of the malformed vessels: macrocystic and microcystic, with the latter being most commonly found in oral lymphangioma [1]. The tongue is the most commonly affected site followed by buccal mucosa, lips and palate [2]. The clinical examination is often sufficient for diagnosing oral lymphangioma; however, there have been cases where histologically confirmed lymphangioma did not align with the clinical diagnosis [2]. Treatment of oral lymphangioma includes conservative surgical excision, laser therapy or sclerotherapy, depending on the size, proximity to adjacent structures and the potential for functional or cosmetic impairment [2].

Alveolar lymphangioma (AL) is a rare type of oral lymphangioma that exclusively affects the alveolar ridge in neonates, accounting for only 2%–4% of all intraoral lymphangiomas with a predilection for Black male infants [3]. It is rarely biopsied as long as it remains indolent and does not interfere with the neonates' feeding function. We present a case of AL of the anterior maxillary alveolar ridge in an 11‐day‐old infant with an unusual appearance, confirmed histologically through immunohistochemical staining. Informed consent for publication was obtained from the parents.

## 2 Case Report

**FIGURE 1:**
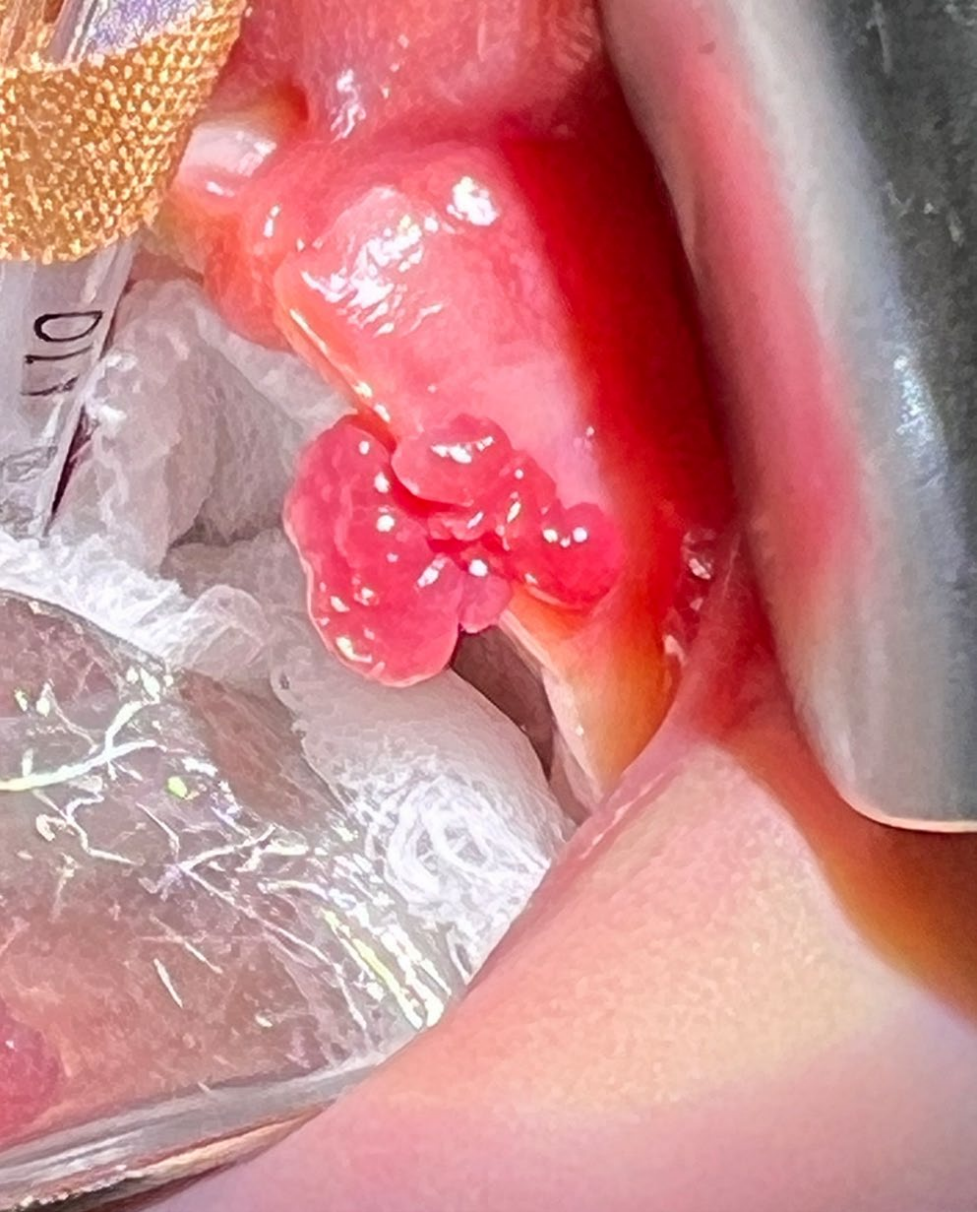
The alveolar lymphangioma presented as a pedunculated lesion exhibiting a lobular/botryoid morphology. The lesion measured approximately 5 mm in widest diameter and displayed a slightly reddish hue compared to the surrounding gingival tissue.

An 11‐day‐old female infant was referred to the dental department for the management of a soft tissue lesion on the alveolar ridge of the upper jaw, provisionally diagnosed as a fibrous epulis. The infant's mother was Samoan and the father was European. Both parents were middle‐aged and had no remarkable medical histories. The infant had an uneventful prenatal period and was delivered through a normal vaginal delivery at full term without complication. She exhibited no apparent health issues. Intraoral examination revealed a 10 × 5 mm exophytic lobular lesion with a slightly reddish colour involving the alveolar ridge in the left maxillary deciduous canine region which intermittently bled after feeding (Figure 1). No other abnormalities were detected intraorally or extraorally.

**FIGURE 2:**
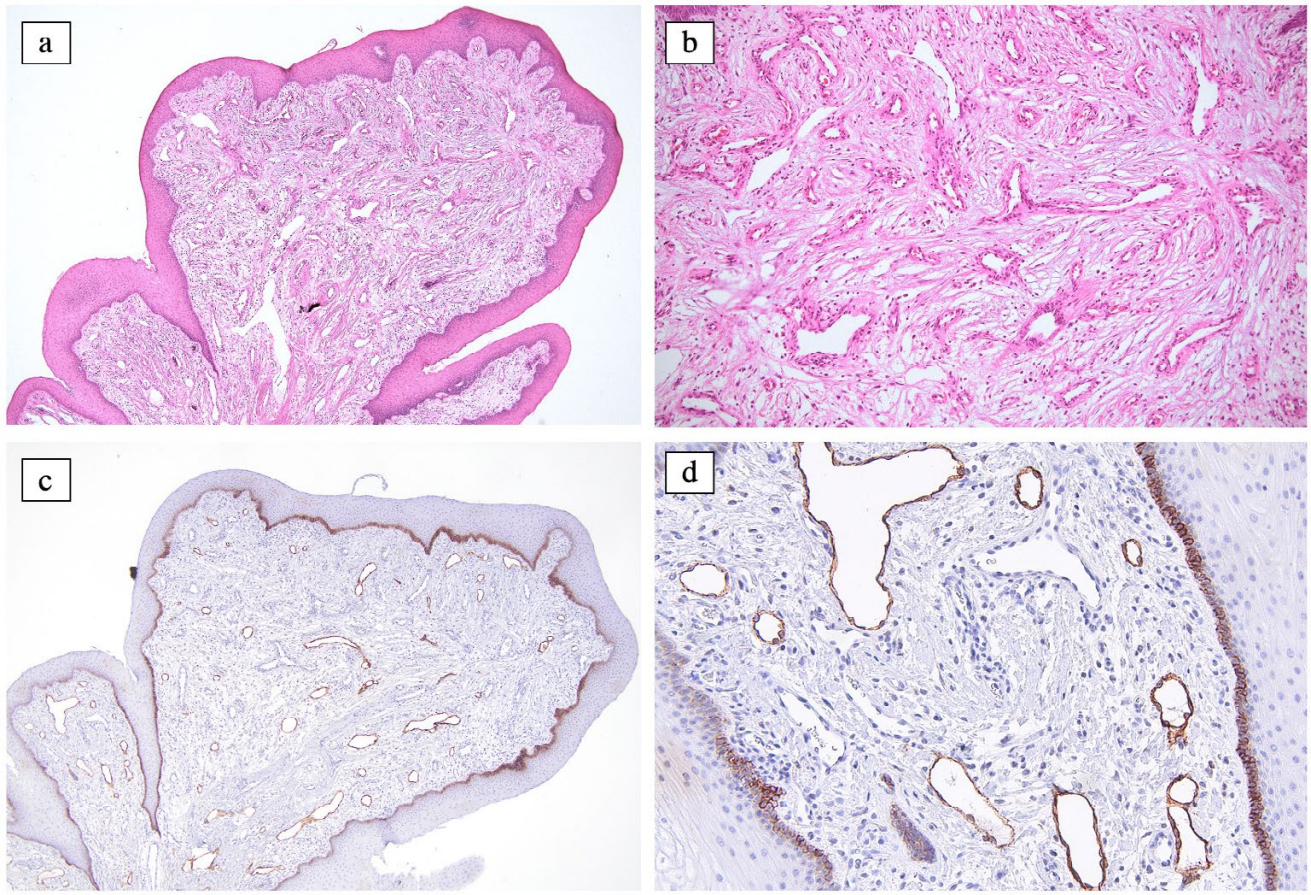
(a) The lesion has polypoid architecture with varying thickness of surface epithelium. Multiple empty lumina can be seen in the background of loose fibrous connective tissue. (b) At higher magnification, the lumen is lined by a single layer of endothelial cells. (c and d) The dilated endothelial lined vessels stained positively with D2‐40 (Podoplanin).

Given the unusual clinical manifestation and the potential for feeding difficulties, such as inadequate palatal seal, an excisional biopsy was performed under general anaesthesia. Histologically, there was an unencapsulated proliferation of endothelial‐lined vessels of varying sizes with a multinodular arrangement in a background of loose, uninflamed fibrous connective tissue, covered by parakeratinised stratified squamous epithelium (Figure 2). The endothelial cells were uniform with no mitotic activity. The vessel lumina were devoid of content. Immunohistochemical staining revealed the majority of luminal endothelial cells were positive to anti‐podoplanin (D2‐40), (Figure 2) leading to the diagnosis of alveolar lymphangioma. The biopsied site healed uneventfully, and the teeth erupted successfully. No recurrence has been observed over a 2‐year follow‐up period.

## 3 Discussion

Since AL was first discovered in 1976, only 64 cases have been reported up to 2023. Of these, only eight included histopathological analysis, and none incorporated immunohistochemical studies [3]. Unlike other lymphangiomas, AL exhibits a notable racial and gender predilection, being frequently observed in black male newborns, and it is sometimes associated with congenital anomalies, including natal teeth, tetralogy of Fallot and congenital heart disease [3]. In our scenario, the female infant was born to a couple of mixed European and Samoan heritage, and the baby was born healthy with no apparent anomalies.

AL typically appears as a bilateral or unilateral bluish, dome‐shaped, fluctuant swelling, usually less than 10 mm in diameter, located on the posterior mandibular and/or maxillary alveolar ridge. The potential differential diagnosis includes alveolar haemangioma, gingival cyst, eruption cyst, mucocoele and congenital epulis [3]. In our case, the AL presented as an isolated, exophytic, slightly reddish pedunculated lesion with a lobular appearance at the alveolar ridge of the left maxillary deciduous canine region, provisionally diagnosed as fibrous epulis. Although most ALs resolve spontaneously without intervention, conservative excisional biopsy should be considered for those with unusual clinical presentation and/or causing functional issues [4], as was observed in our case.

Histologically, AL exhibits a proliferation of uniform, flattened, endothelial‐lined vessels, which may or may not contain acellular eosinophilic material, supported by a loose fibrous connective tissue—findings consistent with our case. Occasional quiescent odontogenic epithelium, interpreted as dental lamina rests, has been observed within the supporting fibrous stroma [4].

This case highlights the occurrence of AL in non‐Black infants and is the first reported on the anterior alveolar ridge of the maxilla as a pedunculated lobular lesion. It is also the first case to be histologically confirmed through immunohistochemistry staining. This case underscores the importance of recognising that, despite the AL's typically benign behaviour, its potential clinical variations and resemblance to other oral lesions, especially neoplasms such as congenital epulis and sarcomas [5], warrant an excisional biopsy for a definitive diagnosis. In addition to reassuring the patient, excision resolves potential aesthetic and feeding issues without leaving any residual defects or scarring.

## Author Contributions

M.A.M.N. – wrote the main manuscript. B.S. – diagnosing oral pathologist, critically reviewed the article. H.M.H. – diagnosing oral pathologist, critically reviewed the article. B.Y. – clinician in charge. A.M.R. – conceptualisation, diagnosing oral pathologist, critically reviewed the article.

## Consent

Consent has been obtained from the patient for publication.

## Conflicts of Interest

The authors declare no conflicts of interest.

## Supporting information


Data S1

## Data Availability

Data sharing is not applicable to this article as no new data were created or analyzed in this study.
